# Structure and process in primary health care for children and spatial distribution of infant mortality

**DOI:** 10.11606/s1518-8787.2024058005527

**Published:** 2024-04-25

**Authors:** Alitéia Santiago Dilélio, Márcio Natividade, Luiz Augusto Facchini, Marcos Pereira, Elaine Tomasi

**Affiliations:** I Universidade Federal de Pelotas Faculdade de Enfermagem Programa de Pós-Graduação em Enfermagem Pelotas RS Brasil Universidade Federal de Pelotas. Faculdade de Enfermagem. Programa de Pós-Graduação em Enfermagem. Pelotas, RS, Brasil.; II Universidade Federal da Bahia Instituto de Saúde Coletiva Programa de Pós-Graduação em Saúde Coletiva Salvador BA Brasil Universidade Federal da Bahia. Instituto de Saúde Coletiva. Programa de Pós-Graduação em Saúde Coletiva. Salvador, BA, Brasil.; III Universidade Federal de Pelotas Faculdade de Medicina Programa de Pós-Graduação em Epidemiologia Pelotas RS Brazil Universidade Federal de Pelotas. Faculdade de Medicina. Programa de Pós-Graduação em Epidemiologia. Pelotas, RS, Brazil.

**Keywords:** Primary Health Care, Spatial Analysis, Assessment of Health Programs and Projects, Assessment of Impact on Health, Infant Mortality

## Abstract

**OBJECTIVE:**

To identify the spatial patterns of the quality of the structure of primary health care services and the teams’ work process and their effects on infant mortality in Brazil.

**METHODS:**

An ecological study of spatial aggregates, using the 5,570 municipalities in Brazil as the unit of analysis. Secondary databases from the *Programa Nacional de Melhoria do Acesso e Qualidade da Atenção Básica* (PMAQ-AB – National Program for Improving Access and Quality of Primary Care), the Mortality Information System (SIM), and the Live Birth Information System (SINASC) were used. In 2018, the infant mortality rate was the outcome of the study, and the exposure variables were the proportion of basic health units (BHU) with adequate structure and work processes. Global and local Moran’s indices were used to evaluate the degree of dependence and spatial autocorrelation. Spatial linear regression was used for data analysis.

**RESULTS:**

In 2018, in Brazil, the infant mortality rate was 12.4/1,000 live births, ranging from 10.6/1,000 and 11.2/1,000 in the South and Southeast, respectively, to 14.1/1,000 and 14.5/1,000 in the Northeast and North regions, respectively. The proportion of teams with an adequate work process (β = −3.13) and the proportion of basic health units with an adequate structure (β = −0.34) were associated with a reduction in the infant mortality rate. Spatial autocorrelation was observed between smoothed mean infant mortality rates and indicators of the structure of primary health care services and the team’s work process, with higher values in the North and Northeast of Brazil.

**CONCLUSIONS:**

There is a relationship between the structure of primary health care services and the teams’ work process with the infant mortality rate. In this sense, investment in the qualification of health care within the scope of primary health care can have an impact on reducing the infant mortality rate and improving child health care.

## INTRODUCTION

Reducing the infant mortality rate (IMR) is one of the Sustainable Development Goals (SDGs), a pact proposed by the United Nations (UN) and signed by 193 countries to promote healthy living and well-being for all. One of the goals is to “reduce neonatal mortality to at least 12 per 1,000 live births and under-five mortality to at least 25 per 1,000 live births” (our translation) by 2030. Children born in more disadvantaged countries are 50 times more likely to die in their first month of life than those born in developed nations^1^.

The IMR indicates the population’s health and socioeconomic development levels and reflects the quality of maternal and child health services. Thus, its reduction is directly related to the improvement of living conditions in populations, becoming a fundamental indicator to guide health actions, programs, and policies^2^. Using this indicator, it is possible to monitor and analyze the availability, use, and effectiveness of health care, identifying the necessary efforts related to prenatal care, childbirth, the newborn, and the child in the first year of life^3^. In Brazil, infant mortality has remained a challenge, despite a significant decline in rates since 2000, going from 25.5 deaths per thousand live births in 1996 to 11.5 per thousand live births in 2020, a reduction of approximately 55%, but with significant regional disparities remaining^3^.

In Brazil, the expansion and consolidation of the Family Health Strategy (FHS) has been strengthening primary health care (PHC), providing increased access to health services with population coverage of 76.1% of PHC and 63. 6% of FHS in 2020, particularly in the least developed regions of the country^6^. This reality has an effect on reducing the IMR by 4.6% for every 10% increase in FHS coverage^7^. The FHS offers actions in maternal and child health with an emphasis on prenatal care, vaccination, breastfeeding, and monitoring the child’s growth and development^8^.

To maintain a strong PHC in the Unified Health System (SUS), it is recommended to “guarantee adequate physical and technological structure, with ambience, comfort and adequate supply of inputs for the operation of basic health units” (our translation)^8^. Structural and work process problems impact the quality of care provided and health indicators, considering the essential attributes of PHC—first contact access, longitudinality, comprehensiveness, and coordination^8^. The work process in PHC is based on forming and strengthening bonds with the population, based on actions such as care coordination, health promotion, multi/interdisciplinarity, home visits, health education, and resolubility of more than 80% of health problems at this level of care^9^.

Territorial inequalities in Brazil are relevant and have a strong impact on morbidity and mortality indicators, especially IMR, such as barriers to access, like the geographical distance between the population and health services^10^. Spatial analysis, by investigating ‘neighborhood effects,’ contributes to the identification of spatial or spatio-temporal clusters, which can help to define health actions, care strategies, and the organization of service provision with a view to protecting vulnerable social groups, focusing on infant mortality, its disparities and risk factors^11^, since it is necessary to know the regional differences between access conditions and the care provided^10,11^.

There is still little research on the effect of determinants related to health services on infant mortality, a gap to which this work aims to contribute by identifying spatial patterns in the quality of the structure of PHC services and the teams’ work process, as well as their effects on infant mortality in Brazil.

## METHODS

### Study design

An ecological, cross-sectional study of spatial aggregates of 5,570 Brazilian municipalities was carried out using secondary data.

### Variables and data source

The outcome was the IMR of the municipalities in 2018, calculated from the absolute number of deaths and live births that year, multiplying the quotient values by one thousand.

Two indicators focused on child health were considered to be the main exposures. The following variables were selected to calculate the proportion of BHU with an adequate structure: existence of an exclusive room for vaccinations; availability of equipment and materials (adult blood pressure equipment, child blood pressure equipment, adult stethoscopes, child stethoscopes, nebulizer equipment, anthropometric scales with a capacity of 150 kg, child scales, child anthropometric rulers, spotlights for gynecological examination, table for gynecological examination, sonars/fetal detector or Pinard, refrigerated chambers or refrigerators exclusively for vaccines, extension cord thermometer, anthropometric measuring rods/tapes); printed material (child health booklet, maternity booklet, and vaccination card); regular vaccination offer; availability of diagnostic tests (rapid syphilis test, rapid pregnancy test, rapid HIV test, rapid Hepatitis B test and rapid Hepatitis C test); and tongue depressors. The following variables were used to calculate the proportion of teams with an adequate work process: family planning actions; registry with the number of high-risk pregnant women in the territory; postpartum consultation within one week of delivery by the team’s doctor and/or nurse; administration of penicillin G benzathine in the health unit; record of child follow-up (up-to-date vaccinations, growth and development, nutritional status, heel prick test, family violence, and accidents); and active search for premature, underweight children, those with delayed childcare consultations and those with delayed vaccination schedules.

Each variable was given a score between 0 and 1 for the absence and presence of the attribute, respectively. The values were then added together, and the indicator represent the proportion of BHU and teams that had all positive values in each municipality, for each of these main exposures.

This information was taken from the *Programa Nacional de Melhoria do Acesso e Qualidade da Atenção Básica* (PMAQ-AB – National Program for Improving Primary Care Access and Quality). The Program had three complete cycles (2012, 2014, and 2018) and was developed in four phases: voluntary adherence by municipalities; self-assessment and monitoring of indicators; external evaluation; and re-contracting. In this article, we worked with the databases from the third cycle of the Program, referring to 2018, available from https://aps.saude.gov.br/ape/pmaq/ciclo3/, encompassing 37,350 PHC teams working in 28,939 BHU throughout Brazil.

### STATISTICAL ANALYSIS

#### Spatial analysis

In 2018, smoothed infant mortality rates were calculated for Brazil and each of its municipalities. The empirical Bayesian smoothing method was used to minimize the instability of crude rates, resulting from small numbers. A weight matrix, or first-order adjacency neighborhood, was built to apply the spatial methods. The spatial distribution patterns of smoothed mean infant mortality rates were identified through visual inspection of thematic maps, built from the distribution of said rates by municipality, using the QGIS® program, version 2.18. The digital mesh of municipalities was made available by the Brazilian Institute of Geography and Statistics (IBGE), in shapefile (shp) format. Using the Geoda® program, version 1.14, and assuming a significance level of 5%, the Global Moran’s Index (GMI) was calculated to assess the presence of spatial autocorrelation between the smoothed mean mortality rates. This indicator was plotted against the spatial lags referring to the distance intervals. The value is between −1 and 1, and the closer to 1, the greater the similarity of attributes between nearby areas. The closer to −1, the less similar they are and, when the value is 0, the attributes are distributed independently and randomly in space. The identification of significant spatial clusters (risk areas) was carried out using Moran’s Local Indicators of Spatial Autocorrelation (Lisa). Lisa indicates spatial clusters of similar values around an observation, comparing the rates of a unit with the mean rate of its neighbors, in addition to testing the statistical significance of this similarity. Values of p < 0.05 are considered significant and indicate spatial autocorrelation.

#### Spatial linear regression

The bivariate and multivariate analyses were carried out using linear regression, with the spatial autoregressive model (SAR – Lag Model), in Geoda® software, version 1.14. In this regression, the spatial dependence component is incorporated into a single parameter in the regression modeling, i.e. spatial autocorrelation is incorporated into the dependent variable. With simultaneous adjustment, the following covariates were considered: family health strategy coverage (e-Gestor – https://egestorab.saude.gov.br/); population size, region, and M-HDI 2010 (IBGE - https://www.ibge.gov.br/); proportion of live births (LB) with seven or more prenatal consultations and proportion of low weight live births (SINASC – https://datasus.saude.gov.br/); and inactivated polio (IPV), pentavalent and triple viral vaccination coverage in the first year of life (SI-PNI – http://pni.datasus.gov.br/).

Data analysis sought to follow a theoretical model based on the social determinants of the health-disease process^12^.

## Ethical aspects

The use of publicly accessible secondary data, without the identification of individuals, does not require assessment by a human research ethics committee, in accordance with National Health Council Resolution No. 466 of December 12, 2012.

## RESULTS

**Figure 1:**
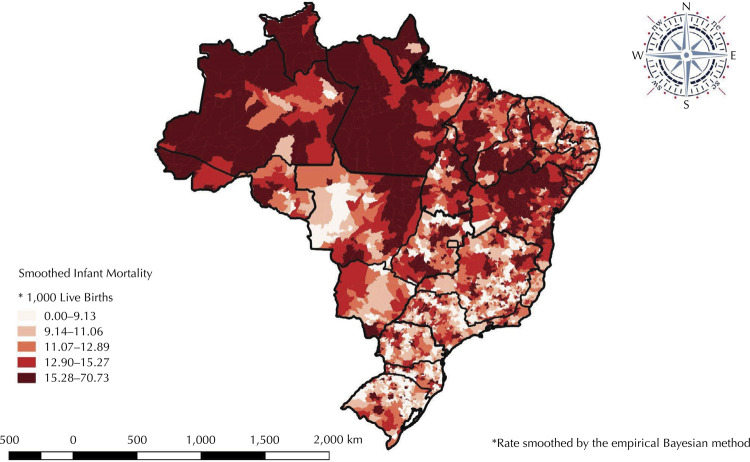
Spatial distribution of the smoothed infant mortality rate*. Brazil, 2018.

**Figure 2:**
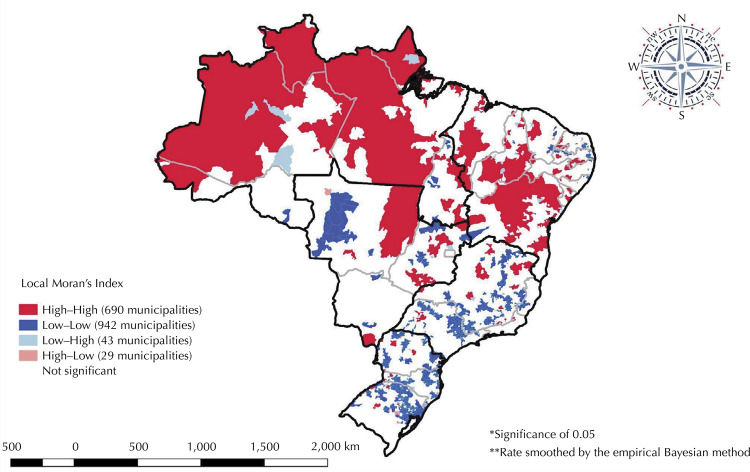
Spatial autocorrelation pattern* of the infant mortality rate**. Brazil, 2018.

In 2018, the IMR in Brazil was 12.4/1,000 LB, ranging from 10.6/1,000 LB and 11.2/1,000 LB in the South and Southeast, respectively, to 14.1/1,000 LB and 14.5/1,000 LB in the Northeast and North regions, respectively. The spatial distribution of the smoothed infant mortality rate showed higher rates in municipalities in the North, Northeast, and Midwest regions (12.9/1,000 LB) (Figure 1). A pattern of spatial autocorrelation of the IMR was observed in the national territory, with a high relationship between the neighboring North, Northeast, and Midwest regions, different from that observed for the South and Southeast regions. Lisa made it possible to identify risk clusters for infant mortality, with a greater concentration in the Northern region of Brazil (Figure 2).

**Figure 3:**
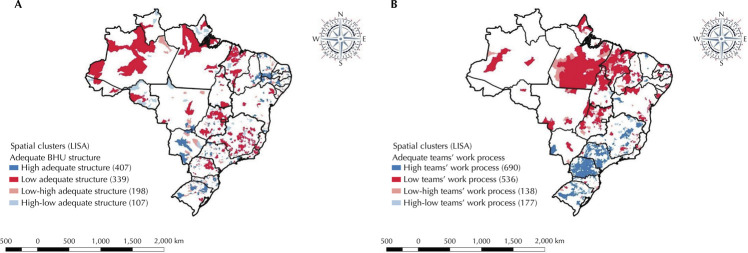
Spatial distribution of the proportion of the UBS structure and the appropriate team work process. Brazil, 2018.

UBS: basic health unit; LISA: Local Moran’s Spatial Autocorrelation Index.

A spatial pattern of service distribution was observed, with structure and teams with an adequate work process, in which Lisa allowed the identification of clusters with smaller proportions for the North and Northeast regions of Brazil (Figure 3).

When evaluating the GMI, spatial autocorrelation was identified between the smoothed average infant mortality rates and the selected indicators, with greater dependence on M-HDI and proportion of LB with seven or more prenatal consultations and lower dependence on IPV and MMR vaccination coverage (Table 1).

**Table 1:** Spatial dependence of the smoothed infant mortality rate and health care indicators. Brazil, 2023.

Indicators	GMI	p-value
Infant mortality rate^a^ (smoothed)	0.511	< 0.05
Proportion of BHU with adequate structure^b^	0.159	< 0.05
Proportion of teams with adequate work process^b^	0.161	< 0.05
Municipal Human Development Index^c^	0.793	< 0.05
Coverage (%) of the Family Health Strategy^d^	0.403	< 0.05
Proportion of live births with seven or more prenatal consultations^e^	0.617	< 0.05
Proportion of low weight live births^e^	0.109	< 0.05
IPV vaccination coverage^f^	0.093	< 0.05
Pentavalent vaccination coverage^f^	0.115	< 0.05
MMR vaccination coverage^f^	0.091	< 0.05

BHU: basic health unit; GMI: Global Moran’s Index.

^a^ Mortality Information System (SIM).

^b^ National Program for Improving Access and Quality of Primary Care (PMAQ-AB).

^c^ Brazilian Institute of Geography and Statistics (IBGE).

^d^ Information and Management of Primary Care (e-Primary Care Manager).

^e^ Information System on Live Births (SINASC).

^f^ National Immunization Program Information System (SI-PNI).

In the bivariate analysis, it was observed that the IMRs were inversely associated and statistically significant with the increase in FHS coverage, IDH-M, the proportion of LB with seven or more prenatal consultations, the proportion of BHU with adequate structure and IPV and pentavalent vaccination coverage. The highest coefficient was recorded for FHS coverage, where, with each percentage increase, on average, there was a reduction of 1.36 deaths/1,000 LB, followed by the proportion of BHU with adequate structure (−1.16). The only variable directly associated with the mortality rate was the proportion of LB with low birth weight, in which with each percentage increase, on average, there was an increase of 0.26 in the IMR for every thousand live births (Table 2).

**Table 2:** Crude and adjusted coefficients of association between the smoothed infant mortality rate and health care indicators. Brazil, 2023.

Indicators	Bivariate SAR model		Multivariate SAR model	
	
	β	p-value	β	p-value
Proportion of BHU with adequate structure^a^	−1.16	< 0.05	−0.34	0.03
Proportion of teams with an adequate work process	−7.41	0.83	−3.13	< 0.05
Municipal Human Development Index^b^	−0.73	< 0.05	−0.51	< 0.05
Coverage (%) of the Family Health Strategy^c^	−1.36	< 0.05	−1.12	< 0.05
Proportion of live births with seven or more prenatal consultations^d^	−0.85	< 0.05	−0.47	< 0.05
Proportion of low weight live births^d^	0.26	< 0.05	0.14	0.05
IPV vaccination coverage^e^	−0.92	< 0.05	−0.15	0.04
Pentavalent vaccine coverage^e^	−1.01	< 0.05	−0.09	0.05
MMR vaccination coverage^e^	−0.16	0.59	−0.07	0.62

BHU: basic health unit; SAR: linear regression with spatial autoregressive model (SAR – Lag Model).

^a^ National Program for Improving Access and Quality of Primary Care (PMAQ-AB).

^b^ Brazilian Institute of Geography and Statistics (IBGE).

^c^ Information and Management of Primary Care (e-Primary Care Manager).

^d^ Live Birth Information System (SINASC).

^e^ National Immunization Program Information System (SI-PNI).

After adjustments, it was clear that despite the decrease in coefficient values, the associations remained significant. The greatest reduction in IMR observed (−3.13) was for the proportion of teams with an adequate work process, since, despite the drop in the adjusted coefficient, it became statistically significant (Table 2).

## DISCUSSION

This study showed the effect of health care indicators on IMR mediated by spatial distribution, with emphasis on the characteristics of PHC services and FHS teams.

The IMR is recognized as a multi-determined indicator, capable of reflecting geographic inequalities, insofar as children under one year of age depend on individual (biological and hereditary) conditions and the environment in which they live^4^. The 2018 IMR showed a spatial distribution with strong inequality between the geopolitical regions of Brazil, with higher risk clusters in the North and Northeast compared to the South and Southeast regions, which has been corroborated by the literature in recent decades, maintaining similar levels in 2017^4^.

A consistent spatial dependence and an inverse correlation were identified with most of the indicators investigated, except for the proportion of low weight live births, which showed a direct relationship, demonstrating that infant mortality is quite sensitive to regional inequalities and reflects on other indicators. This finding may occur due to chronic situations of social and health deprivation, with lack of access and low coverage of services, including prenatal care, in addition to a shortage of professionals. According to Faria^4^, this scenario reinforces the “social and geographic determination of infant mortality, since social and historical processes determine territorial profiles that are more or less favorable to the survival of children in their first year of life.”

The use of georeferencing in the context of health has advanced over the decades, from the investigation conducted by John Snow to identify the source of the 1854 cholera outbreak in London, to the recent COVID-19 pandemic^13^. Different spatial information systems have helped to highlight disparities in the territory in various applications, such as epidemiological surveillance of communicable diseases, incidence of morbid events and delimitation of risk areas for mortality^14,15^. Another useful application is to select areas of intervention and indicators that are more sensitive to strategic health actions, so that progress can be made in reducing infant mortality, recognizing historical regional inequalities, whether social, cultural, economic or political^4^.

One of the consequences of this situation is the maintenance of a pattern of unequal distribution of supply and access to services, with differences between Brazil’s least and most developed regions. In addition to these macro-regional inequalities, differences in the service provision can be observed within the same municipality, between municipalities and even between the states of the federation^14^.

This study found that municipalities with a higher HDI-M, i.e. better socioeconomic status, had lower IMR. Martins et al.^16^observed that despite the improvement in the M-HDI in all Brazilian regions, marked regional differences remained in 2010 as compared to 2000. The authors emphasized that there was greater variability in the IMR in regions with worse living conditions, demonstrating that both indices are sensitive and capable of reflecting territorial differences, since in the regions where there was an increase in the HDI-M there was a reduction in the IMR^16^. It is important to understand that this index reflects the reality of macro-regions and can conceal areas of low development and social vulnerability. High IMRs can reflect socioeconomic inequalities and public policy deficits^17^.

In Brazil, the FHS is the main PHC model and has stood out as one of the main pillars of the SUS. It is characterized by a set of health promotion, disease prevention and rehabilitation actions carried out by a multidisciplinary team in a defined territory. The FHS guarantees comprehensive and free care to all people “according to their needs and the demands of the territory, considering the determinants and conditioning factors of health” (our translation) ^18^.

Among PHC’s programmatic actions is the Childcare Program, which monitors children’s development and growth, as well as timely vaccinations, and prevents diseases in the first years of life, such as diarrhea and respiratory infections, which are still responsible for a large proportion of infant deaths^18^.

A study carried out with information from the SIM between 2000 to 2015 identified that the main causes of infant death were considered preventable by primary care (conditions originating in the perinatal period, infectious and parasitic diseases, and diseases of the respiratory system), with the exception of congenital malformations, deformities, and chromosomal anomalies, which are prominent in the Southeast region of the country^19^. Characteristics such as low average state income, low birth weight, fewer prenatal consultations, and increased fertility rates are associated with higher IMR in the country, showing a direct relationship with some PHC attributes^20^.

Evidence of the positive effects of the expansion of the FHS on access, quality of services, a reduction in standardized hospitalization rates for primary care- sensitive conditions and a consequent decline in infant mortality in Brazil over the last few decades confirms its effectiveness in organizing PHC in Brazil^9,21^. Observing the data from 2013 to 2019 from the National Health Surveys (PNS), it is clear that the FHS remains the main PHC model in the SUS, with strong role in favor of equity^22^.

Recent initiatives by the Ministry of Health have encouraged the evaluation of health facilities receiving financial resources in terms of structure, process, and results, such as PMAQ-AB in 2011. Examples include the 2013 program for requalifying the Infrastructure of Basic Health Units of the country (*Requalifica UBS*) and the 2015 *Programa Nacional de Avaliação de Serviços de Saúde* (PNASS – National Health Services Assessment Program). Since Donabedian’s research^23^, it is understood that health services that have an adequate structure and teams that follow the protocols and principles of its care model tend to produce better results in the health situation of the enrolled population. This understanding can support decision-making to improve the population’s health and reduce inequalities^24^. In general, teams have more autonomy to modify their work process than to interfere in improving infrastructure, since this involves financial resources that are not always available^24,25^.

It was with this intention that the analyses in this work prioritized these characteristics of services and teams as essential to mitigate the effect of contextual inequalities and their effects on more proximal determinants of mortality, such as prenatal care, low birth weight, and vaccination coverage^12^.

Similar to the findings of this study, Hatisuka et al.^26^also identified a direct relationship between the lowest IMRs and units with a good performance rating in the PMAQ evaluation, although the rating used was not directly related to the maternal and child health care, like the exposures selected in these analyses.

Studies carried out in Brazil in recent years continue to demonstrate a higher risk of infant mortality among children who have low birth weight^27^and those whose mothers had fewer prenatal consultations^17^.

Intrauterine growth retardation, cesarean section births and prematurity lead to a greater occurrence of low birth weight, contributing to worsening the newborn’s health and making them more vulnerable to diseases and infections^28^. As recommended by the Ministry of Health, adequate prenatal care allows for the timely diagnosis and treatment of risk factors and complications during pregnancy, reducing or modifying risk behaviors to alleviate the morbidity and mortality of the mother and fetus^29^. It is necessary to guarantee prevention and protection measures, such as starting monitoring until the 12th week of pregnancy, carrying out at least six consultations, two routine laboratory tests, and rapid tests for HIV, syphilis, and hepatitis B and C^30^.

Both birth weight and prenatal consultations are directly related to the quality of care during pregnancy and childbirth and may reflect socioeconomic status and maternal morbidity, in addition to maternal behaviors in relation to health care^31^. The findings of this study confirm the importance of PHC in monitoring and adherence to prenatal care, strengthening bonds with users, and family planning. Despite advances in health actions, programs, and policies that encourage users to adhere to health care and combat poverty, such as Bolsa Família^32^, investment is needed to qualify the teams’ work process and improve the service infrastructure, such as PMAQ^26^.

Still within the scope of PHC, another important strategy for preventing child mortality refers to immunizations. The *Programa Nacional de Imunizações* (PNI – National Immunization Program), approved in 1973, seeks to provide a better quality of life for the population through disease prevention. Vaccines stimulate the immune system, protecting one’s health and preventing disabilities and deaths from preventable diseases, such as polio, diphtheria, tetanus, whooping cough, hepatitis B, *haemophilus influenza* type b, measles, mumps and rubella, among others. The reduction in vaccination coverage has the potential for the emergence of epidemics and an increase in infant mortality^33,34^.

Immunizations can be considered one of the main adjuvant technologies for reducing child mortality, as they contributed to the eradication of some diseases, also acting to reduce the severity of symptoms and contributing to an increase in life expectancy. Since the discovery of the treatment for smallpox, in 1796, by Edward Jenner, until the advent of the vaccine against COVID-19, the efficacy, effectiveness, safety, and protection of immunizers at an individual and collective level have been observed^35^.

The limitations of this study include the use of secondary databases, without the possibility of analyzing other factors associated with infant mortality, such as nutritional status, illness, and use of health services.

This study showed the relationship between the structure of PHC services and the teams’ work process and IMR, regardless of individual factors, such as prenatal consultations, birth weight, and vaccination status. In this sense, investment in the qualification of health care within the scope of PHC can have an impact on reducing IMR and improving child health care.

For the organization of PHC and the planning of health actions, it is essential to consider regional differences, due to cultural, economic and social heterogeneity, in order to qualify the care provided, identify risk areas and select indicators that are more sensitive to strategic actions.

Even in the face of the decline in IMR over the last few decades, the effect of the COVID-19 pandemic may result in a worsening of health indicators, given the possible reduction in the supply of PHC programmatic actions to meet the demand in dealing with the pandemic.
