# Supplementary material for: Structure and process in primary health care for children and spatial distribution of infant mortality
Source: Rev Saude Publica. 2024 Apr 25;58:21. doi: 10.11606/s1518-8787.2024058005527 (PMC11090610; doi:10.11606/s1518-8787.2024058005527)
Supplement: Translation

# Translation: Estrutura e processo na atenção primária à saúde das crianças e distribuição espacial da mortalidade infantil

**Authors:** Alitéia Santiago Dilélio, Márcio Natividade, Luiz Augusto Facchini, Marcos Pereira, Elaine Tomasi

**Affiliations:** I Brasil Universidade Federal de Pelotas. Faculdade de Enfermagem. Programa de Pós-Graduação em Enfermagem. Pelotas, RS, Brasil.; II Brasil Universidade Federal da Bahia. Instituto de Saúde Coletiva. Programa de Pós-Graduação em Saúde Coletiva. Salvador, BA, Brasil.; III Brasil Universidade Federal de Pelotas. Faculdade de Medicina. Programa de Pós-Graduação em Epidemiologia. Pelotas, RS, Brasil.

**Keywords:** Atenção Primária à Saúde, Análise Espacial, Avaliação de Programas e Projetos de Saúde, Avaliação do Impacto na Saúde, Mortalidade Infantil

## Abstract

**OBJETIVO:**

Identificar os padrões espaciais da qualidade da estrutura dos serviços de atenção primária à saúde e do processo de trabalho das equipes e seus efeitos na mortalidade infantil no Brasil.

**MÉTODOS:**

Estudo ecológico de agregados espaciais, empregando como unidade de análise os 5.570 municípios do Brasil. Foram utilizados bancos de dados secundários do Programa Nacional de Melhoria do Acesso e Qualidade da Atenção Básica (PMAQ-AB), do Sistema de Informação de Mortalidade (SIM) e do Sistema de Informações de Nascidos Vivos (SINASC). Em 2018, a taxa de mortalidade infantil foi o desfecho do estudo, e as variáveis de exposição foram a proporção de unidade básica de saúde (UBS) com estrutura e processo de trabalho adequados. Os índices de Moran global e local foram usados para avaliar o grau de dependência e a autocorrelação espacial. Utilizou-se regressão linear espacial para análise de dados.

**RESULTADOS:**

Em 2018, no Brasil, a taxa de mortalidade infantil foi de 12,4/1.000 nascidos vivos, variando de 10,6/1.000 e 11,2/1.000 no Sul e no Sudeste, respectivamente, até 14,1/1.000 e 14,5/1.000, nas regiões Nordeste e Norte, respectivamente. A proporção de equipes com processo de trabalho adequado (β = - 3,13) e a de unidades básicas de saúde com estrutura adequada (β = - 0,34) foram associadas à redução da taxa de mortalidade infantil. Observou-se autocorrelação espacial entre as taxas de mortalidade infantil médias suavizadas e indicadores da estrutura dos serviços de atenção primária à saúde e do processo de trabalho das equipes, com valores mais elevados no Norte e no Nordeste do Brasil.

**CONCLUSÕES:**

Existe relação entre a estrutura dos serviços de atenção primária à saúde e o processo de trabalho das equipes com a taxa de mortalidade infantil. Neste sentido, o investimento na qualificação da atenção à saúde no âmbito da atenção primária à saúde pode impactar na redução da taxa de mortalidade infantil e na melhoria da atenção à saúde infantil.

## INTRODUÇÃO

A redução da taxa de mortalidade infantil (TMI) compõe um dos Objetivos de Desenvolvimento Sustentável (ODS), pacto proposto pela Organização das Nações Unidas (ONU), assinado por 193 países, em prol de uma vida saudável e da promoção do bem-estar para todos. Entre as metas, destaca-se “reduzir a mortalidade neonatal para pelo menos 12 por 1.000 nascidos vivos e a mortalidade de crianças menores de 5 anos para pelo menos 25 por 1.000 nascidos vivos”, até 2030. Crianças que nascem em países mais desfavorecidos têm probabilidade 50 vezes maior de morrer no primeiro mês de vida do que as nascidas em nações desenvolvidas^1^.

A TMI indica os níveis de saúde e desenvolvimento socioeconômico da população e reflete a qualidade dos serviços de saúde materno-infantil. Assim, sua redução guarda relação direta com a melhoria das condições de vida nas populações, tornando-se um indicador fundamental para orientar ações, programas e políticas de saúde^2^. A partir deste indicador, é possível monitorar e analisar a disponibilidade, a utilização e a efetividade dos cuidados de saúde, identificando os esforços necessários relacionados à atenção pré-natal, ao parto, ao recém-nascido e à criança no primeiro ano de vida^3^. No Brasil, a mortalidade infantil tem se perpetuado como um desafio, mesmo diante de um declínio importante das taxas a partir de 2000, indo de 25,5 óbitos por mil nascidos vivos, em 1996, para 11,5 por mil nascidos vivos, em 2020, uma redução de aproximadamente 55%, mas com a manutenção de importantes disparidades regionais^3^.

No Brasil, a expansão e a consolidação da Estratégia de Saúde da Família (ESF) vem fortalecendo a atenção primária à saúde (APS), propiciando a ampliação do acesso aos serviços de saúde com cobertura populacional de 76,1% de APS e 63,6% de ESF em 2020, particularmente nas regiões menos desenvolvidas do país^6^. Esta realidade tem efeito na redução da TMI, de 4,6% para cada 10% de aumento da cobertura da ESF^7^. A ESF oferta ações em saúde materno-infantil com ênfase no pré-natal, na vacinação, no aleitamento materno e no acompanhamento do crescimento e do desenvolvimento^8^ da criança.

Para a manutenção de uma APS forte no Sistema Único de Saúde (SUS), recomenda-se “garantir estrutura física e tecnológica adequadas, com ambiência, conforto e fornecimento adequado de insumos para o funcionamento das unidades básicas de saúde”. Problemas estruturais e de processo de trabalho impactam na qualidade da assistência prestada e nos indicadores de saúde, considerando os atributos essenciais da APS – acesso de primeiro contato, longitudinalidade, integralidade e coordenação^8^. O processo de trabalho na APS baseia-se na formação e no fortalecimento de vínculos com a população, a partir de ações como coordenação do cuidado, promoção da saúde, multi/interdisciplinaridade, visitas domiciliares, educação em saúde e resolubilidade de mais de 80% dos problemas de saúde nesse nível de atenção^9^.

As desigualdades territoriais no Brasil são relevantes e impactam fortemente nos indicadores de morbimortalidade, principalmente na TMI, a exemplo das barreiras de acesso, como a distância geográfica entre a população e os serviços de saúde^10^. A análise espacial, ao investigar os ‘efeitos de vizinhança’, contribui para a identificação de aglomerados espaciais ou espaço-temporais, que podem auxiliar na definição de ações de saúde, estratégias de assistência e organização da oferta de serviços com vistas à proteção de grupos sociais vulneráveis, focando na mortalidade infantil, suas disparidades e fatores de risco^11^, uma vez que é preciso conhecer as diferenças regionais existentes entre as condições de acesso e a assistência prestada^10,11^.

Ainda são escassas as investigações sobre o efeito de determinantes relacionados aos serviços de saúde sobre a mortalidade infantil, lacuna para a qual este trabalho pretende contribuir ao identificar padrões espaciais da qualidade da estrutura dos serviços de APS e do processo de trabalho das equipes, bem como seus efeitos na mortalidade infantil no Brasil.

## MÉTODOS

### Desenho de estudo

Realizou-se um estudo ecológico, de abordagem transversal, de agregados espaciais de 5.570 municípios do Brasil, com dados secundários.

### Variáveis e fonte dos dados

Tomou-se como desfecho as TMI dos municípios em 2018, calculadas a partir do número absoluto de óbitos e de nascidos vivos neste ano, multiplicando-se os valores do quociente por mil.

Dois indicadores voltados para a saúde infantil foram considerados exposições principais. Para compor a proporção de UBS com estrutura adequada, foram selecionadas as seguintes variáveis: existência de sala exclusiva para vacina; disponibilidade de equipamentos e materiais (aparelhos de pressão adulto, aparelhos de pressão infantil, estetoscópios adulto, estetoscópios infantil, aparelho de nebulização, balanças antropométricas com capacidade de 150 kg, balanças infantis, réguas antropométricas infantil, focos de luz para exame ginecológico, mesa para exame ginecológico, sonares/detector fetal ou pinard, câmaras refrigeradas ou geladeiras exclusivas para vacina, termômetro de cabo extensor, trenas/fitas antropométricas); material impresso (caderneta de saúde da criança, caderneta da gestante e cartão de vacinação); oferta regular de vacinação; disponibilidade de testes diagnósticos (teste rápido de sífilis, teste rápido de gravidez, teste rápido de HIV, teste rápido de Hepatite B e teste rápido de Hepatite C); e abaixador de língua. Para compor a proporção de equipes com processo de trabalho adequado, foram utilizadas as seguintes variáveis: ações de planejamento familiar; registro com o número de gestantes de alto risco no território; consulta de puerpério até uma semana após o parto pelo médico e/ou enfermeiro da equipe; aplicação da penicilina G benzatina na unidade de saúde; registro do acompanhamento das crianças (vacinação em dia, crescimento e desenvolvimento, estado nutricional, teste do pezinho, violência familiar e acidentes); e realização de busca ativa de crianças prematuras, com baixo peso, com consulta de puericultura atrasada e com calendário vacinal atrasado.

Cada variável recebeu uma pontuação entre 0 e 1, para ausência e presença do atributo, respectivamente. Em seguida, foram somados os valores, sendo o indicador representativo da proporção de UBS e equipes que apresentaram todos os valores positivos em cada município, para cada uma destas exposições principais.

Estas informações foram retiradas do Programa Nacional de Melhoria do Acesso e Qualidade da Atenção Básica (PMAQ-AB). O Programa teve três ciclos completos (2012, 2014 e 2018) e desenvolveu-se em quatro fases: adesão voluntária dos municípios; autoavaliação e monitoramento de indicadores; avaliação externa; e recontratualização. Neste artigo, trabalhou-se com os bancos de dados do terceiro ciclo do Programa, referentes a 2018, disponíveis em https://aps.saude.gov.br/ape/pmaq/ciclo3/, abrangendo 37.350 equipes de APS atuantes em 28.939 UBS de todo o Brasil.

## ANÁLISE ESTATÍSTICA

### Análise espacial

Em 2018, as taxas suavizadas de mortalidade infantil foram calculadas para o Brasil e para cada um de seus municípios. Visando minimizar a instabilidade das taxas brutas, resultantes de pequenos números, aplicou-se o método de suavização bayesiano empírico. Foi construída uma matriz de peso, ou vizinhança por adjacência de primeira ordem, para aplicar os métodos espaciais. A identificação dos padrões de distribuição espacial das taxas médias suavizadas da mortalidade infantil foi realizada mediante inspeção visual de mapas temáticos, construídos a partir da distribuição das referidas taxas por municípios, com o emprego do programa QGIS®, versão 2.18. A malha digital dos municípios foi disponibilizada pelo Instituto Brasileiro de Geografia e Estatística (IBGE), em formato *shapefile* (shp). Utilizando-se o programa Geoda®, versão 1.14, e admitindo-se um nível de significância de 5%, foi calculado o Índice Global de Moran (IGM) para avaliar a presença de autocorrelação espacial entre as taxas médias suavizadas de mortalidade. Esse indicador foi plotado contra as defasagens espaciais referentes aos intervalos de distância. O valor está entre – 1 e 1, e quanto mais próximo de 1, maior a similaridade de atributos entre áreas próximas. Quanto mais próximo de – 1, menos semelhantes eles são e, quando o valor é 0, os atributos são distribuídos de forma independente e aleatória no espaço. A identificação dos *clusters* espaciais significantes (áreas de risco) foi realizada a partir do Índice Local de Autocorrelação Espacial de Moran (Lisa). O Lisa indica os aglomerados espaciais de valores semelhantes em torno de uma observação, comparando as taxas de uma unidade com a taxa média de seus vizinhos, além de testar a significância estatística dessa similaridade. Valores de p < 0,05 são considerados significativos e indicam autocorrelação espacial.

### Regressão linear espacial

As análises bivariadas e multivariadas foram realizadas por meio de regressão linear, com o modelo espacial autorregressivo (SAR – Lag Model), no software Geoda®, versão 1.14. Nesta regressão, o componente da dependência espacial é incorporado num único parâmetro na modelagem regressiva, ou seja, a autocorrelação espacial é incorporada na variável dependente. Com ajuste simultâneo, considerou-se as seguintes covariáveis: cobertura de estratégia de saúde da família (e-Gestor – https://egestorab.saude.gov.br/); porte populacional, região e IDH-M 2010 (IBGE – https://www.ibge.gov.br/); proporção de nascidos vivos (NV) com sete ou mais consultas de pré-natal e proporção de nascidos vivos com baixo peso ao nascer (SINASC – https://datasus.saude.gov.br/); e coberturas vacinais de pólio inativada (VIP), pentavalente e tríplice viral no primeiro ano de vida (SI-PNI – http://pni.datasus.gov.br/).

A análise dos dados procurou seguir um modelo teórico baseado nos determinantes sociais do processo saúde-doença^12^.

### Aspectos éticos

A utilização de dados secundários, de acesso público, sem identificação de pessoas, dispensa a apreciação por comitê de ética em pesquisa com seres humanos, em conformidade com a Resolução do Conselho Nacional de Saúde, nº 466, de 12 de dezembro de 2012.

## RESULTADOS

Em 2018, a TMI, no Brasil, foi de 12,4/1.000 NV, variando de 10,6/1.000 NV e 11,2/1.000 NV no Sul e no Sudeste, respectivamente, até 14,1/1.000 NV e 14,5/1.000 NV, nas regiões Nordeste e Norte, respectivamente. A distribuição espacial da taxa suavizada de mortalidade infantil evidenciou maiores taxas em municípios das regiões Norte, Nordeste e Centro-Oeste (12,9/1.000 NV) (Figura 1). Observou-se um padrão de autocorrelação espacial da TMI no território nacional, com uma relação alta entre as regiões vizinhas Norte, Nordeste e Centro-Oeste, diferente do observado para as regiões Sul e Sudeste. O Lisa permitiu identificar *clusters* de risco para mortalidade infantil, com maior concentração na região Norte do Brasil (Figura 2).

**Figura 1:** Distribuição espacial da taxa de mortalidade infantil suavizada*. Brasil, 2018.

**Figura 2:** Padrão de autocorrelação espacial* da taxa de mortalidade infantil**. Brasil, 2018.

Observou-se um padrão espacial da distribuição de serviços, com estrutura e equipes com processo de trabalho adequados, no qual o Lisa permitiu identificar *clusters* com menores proporções para as regiões Norte e Nordeste do Brasil (Figura 3).

**Figura 3:** Distribuição espacial da proporção da estrutura da UBS e do processo de trabalho das equipes adequado. Brasil, 2018.

UBS: unidade básica de saúde; LISA: Índice Local de Autocorrelação Espacial de Moran.

Ao avaliar o IGM, identificou-se a autocorrelação espacial entre as taxas médias suavizadas de mortalidade infantil e os indicadores selecionados, com maior dependência para IDH-M e proporção de NV com sete ou mais consultas de pré-natal e menor dependência para as coberturas vacinais de VIP e de tríplice viral (Tabela 1).

**Tabela 1:** Dependência espacial da taxa de mortalidade infantil suavizada e indicadores de atenção à saúde. Brasil, 2023.

Indicadores	IGM	p-valor
Taxa de mortalidade infantil^a^ (suavizada)	0,511	< 0,05
Proporção de UBS com estrutura adequada^b^	0,159	< 0,05
Proporção de equipes com processo de trabalho adequado^b^	0,161	< 0,05
Índice de Desenvolvimento Humano Municipal^c^	0,793	< 0,05
Cobertura (%) da Estratégia Saúde da Família^d^	0,403	< 0,05
Proporção de nascidos vivos com sete ou mais consultas de pré-natal^e^	0,617	< 0,05
Proporção de nascidos vivos com baixo peso ao nascer^e^	0,109	< 0,05
Cobertura vacinal VIP^f^	0,093	< 0,05
Cobertura vacinal pentavalente^f^	0,115	< 0,05
Cobertura vacinal tríplice viral^f^	0,091	< 0,05

UBS: unidade básica de saúde; IGM: Índice Global de Moran.

^a^Sistema de Informação sobre Mortalidade (SIM).

^b^Programa Nacional de Melhoria do Acesso e da Qualidade da Atenção Básica (PMAQ-AB).

^c^Instituto Brasileiro de Geografia e Estatística (IBGE).

^d^Informação e Gestão da Atenção Básica (e-Gestor Atenção Básica).

^e^Sistema de Informações sobre Nascidos Vivos (SINASC).

^f^Sistema de Informação do Programa Nacional de Imunizações (SI-PNI).

Na análise bivariada, observou-se que as TMI estiveram inversamente associadas e estatisticamente significativas ao aumento da cobertura de ESF, do IDH-M, da proporção de NV com sete ou mais consultas de pré-natal, da proporção de UBS com estrutura adequada e das coberturas vacinais de VIP e pentavalente. O maior coeficiente foi registrado para a cobertura de ESF, em que, a cada aumento percentual, em média, houve redução de 1,36 mortes/1.000 NV, seguido da proporção de UBS com estrutura adequada (- 1,16). A única variável associada diretamente com a taxa de mortalidade foi a proporção de NV com baixo peso ao nascer, em que a cada aumento percentual, em média, houve o aumento de 0,26 na TMI a cada mil nascidos vivos (Tabela 2).

**Tabela 2:** Coeficientes brutos e ajustados de associação entre taxa suavizada de mortalidade infantil e indicadores de atenção à saúde. Brasil, 2023.

Indicadores	Modelo SAR bivariado		Modelo SAR multivariado	
	
	β	p-valor	β	p-valor
Proporção de UBS com estrutura adequada^a^	- 1,16	< 0,05	- 0,34	0,03
Proporção de equipes com processo de trabalho adequado^a^	- 7,41	0,83	- 3,13	< 0,05
Índice de Desenvolvimento Humano Municipal^b^	- 0,73	< 0,05	- 0,51	< 0,05
Cobertura (%) da Estratégia Saúde da Família^c^	- 1,36	< 0,05	- 1,12	< 0,05
Proporção de nascidos vivos com sete ou mais consultas de pré-natal^d^	- 0,85	< 0,05	- 0,47	< 0,05
Proporção de nascidos vivos com baixo peso ao nascer^d^	0,26	< 0,05	0,14	0,05
Cobertura vacinal VIP^e^	- 0,92	< 0,05	- 0,15	0,04
Cobertura vacinal pentavalente^e^	- 1,01	< 0,05	- 0,09	0,05
Cobertura vacinal tríplice viral^e^	- 0,16	0,59	- 0,07	0,62

UBS: unidade básica de saúde; SAR: regressão linear com modelo espacial autoregressivo (SAR – Lag Model).

^a^Programa Nacional de Melhoria do Acesso e da Qualidade da Atenção Básica (PMAQ-AB).

^b^Instituto Brasileiro de Geografia e Estatística (IBGE).

^c^Informação e Gestão da Atenção Básica (e-Gestor Atenção Básica).

^d^Sistema de Informações sobre Nascidos Vivos (SINASC).

^e^Sistema de Informação do Programa Nacional de Imunizações (SI-PNI).

Após ajustes, evidenciou-se que, apesar da diminuição dos valores dos coeficientes, as associações se mantiveram significativas. A maior redução da TMI observada (- 3,13) foi para a proporção de equipes com processo de trabalho adequado, pois, apesar da queda no coeficiente ajustado, tornou-se estatisticamente significativo (Tabela 2).

## DISCUSSÃO

Neste estudo, evidenciou-se o efeito de indicadores de atenção à saúde sobre a TMI mediado pela distribuição espacial, com destaque para as características dos serviços de APS e das equipes de ESF.

A TMI é reconhecidamente um indicador de determinação múltipla, capaz de refletir as desigualdades geográficas, na medida em que crianças menores de um ano de idade dependem das condições individuais (biológicas e hereditárias) e do meio em que vivem^4^. A TMI de 2018 apresentou uma distribuição espacial com forte desigualdade entre as regiões geopolíticas do Brasil, observando-se *clusters* de maior risco nas regiões Norte e Nordeste, comparadas às regiões Sul e Sudeste, o que tem sido corroborado pela literatura nas últimas décadas, mantendo níveis similares em 2017^4^.

Identificou-se uma consistente dependência espacial e uma correlação inversa com a maioria dos indicadores investigados, à exceção da proporção de nascidos vivos com baixo peso, que apresentou uma relação direta, demonstrando que a mortalidade infantil é bastante sensível às desigualdades regionais e reflete em outros indicadores. Este achado pode ocorrer devido a situações crônicas de privação social e de saúde, com falta de acesso e baixa cobertura dos serviços, incluindo pré-natal, além de escassez de profissionais. De acordo com Faria^4^, este cenário reforça a “determinação social e geográfica da mortalidade infantil, uma vez que processos sociais e históricos determinam perfis territoriais mais ou menos favoráveis à sobrevida de crianças no seu primeiro ano de vida”.

O uso do georreferenciamento no contexto da saúde tem avançado ao longo das décadas, desde a investigação conduzida por John Snow para identificar a fonte causadora do surto de cólera de 1854, em Londres, até a recente pandemia do covid-19^13^. Diferentes sistemas de informações espaciais têm auxiliado a evidenciar disparidades no território em diversas aplicações, como vigilância epidemiológica de doenças transmissíveis, incidência de eventos mórbidos e delimitação de áreas de risco para mortalidade^14,15^. Outra utilidade a ser destacada é a de selecionar áreas de intervenção e indicadores mais sensíveis a ações estratégicas de saúde, que permitam o avanço da redução da mortalidade infantil, reconhecendo as desigualdades regionais históricas, sejam elas sociais, culturais, econômicas ou políticas^4^.

Uma das consequências desta conjuntura é a manutenção de um padrão de distribuição desigual da oferta e do acesso aos serviços, com diferenciais entre as regiões menos e mais desenvolvidas do Brasil. E, para além destas desigualdades macrorregionais, as diferenças na oferta de serviços podem ser observadas no interior de um mesmo município, entre municípios e mesmo entre os estados da federação^14^.

Neste estudo, identificou-se que municípios com maior IDH-M, ou seja, com melhor situação socioeconômica, apresentaram menores TMI. Martins et al.^16^ observaram que, apesar da melhora do IDH-M em todas as regiões brasileiras, mantiveram-se diferenças regionais marcantes, em 2010, se comparadas a 2000. Os autores ressaltaram que houve maior variabilidade na TMI nas regiões com piores condições de vida, demonstrando que ambos os índices são sensíveis e capazes de refletir diferenças territoriais, pois nas regiões em que houve aumento do IDH-M observou-se a redução da TMI^16^. É importante compreender que esse índice reflete a realidade das macrorregiões, podendo encobrir áreas de baixo desenvolvimento e vulnerabilidade social. Altas TMI podem refletir desigualdades socioeconômicas e déficit de políticas públicas^17^.

No Brasil, a ESF é o principal modelo de APS e tem se destacado como um dos principais pilares do SUS, caracterizando-se por um conjunto de ações de promoção da saúde, prevenção de agravos e reabilitação, realizado por equipe multiprofissional, em território definido. A ESF garante cuidados integrais e gratuitos a todas as pessoas “de acordo com suas necessidades e demandas do território, considerando os determinantes e condicionantes de saúde”^18^.

Entre as ações programáticas da APS, destaca-se o Programa de Puericultura, que acompanha o desenvolvimento e o crescimento infantil, assim como vacinação oportuna, e previne doenças nos primeiros anos de vida, como a diarreia e as infecções respiratórias, ainda responsáveis por grande parte dos óbitos infantis^18^.

Estudo realizado com informações do SIM no período de 2000 a 2015 identificou que as principais causas de óbito infantil foram consideradas evitáveis pela atenção básica (afecções originadas no período perinatal, doenças infecciosas e parasitárias e doenças do aparelho respiratório), à exceção das malformações congênitas, deformidades e anomalias cromossômicas que se destacam na região Sudeste do país^19^. Características como baixa renda média estadual, baixo peso ao nascer, menor número de consultas de pré-natal e aumento da taxa de fecundidade apresentam-se associados à maior TMI no país, evidenciando relação direta com alguns atributos da APS^20^.

Evidências sobre os efeitos positivos da expansão da ESF no acesso, na qualidade dos serviços, na redução das taxas padronizadas de internações por condições sensíveis à atenção básica e consequente declínio da mortalidade infantil no Brasil, ao longo das últimas décadas, ratificam sua efetividade para a organização da APS no Brasil^9,21^. Observando os dados de 2013 a 2019 das Pesquisas Nacionais de Saúde (PNS), compreende-se que a ESF permanece como principal modelo de APS no SUS, com forte atuação em prol da equidade^22^.

Recentes iniciativas do Ministério da Saúde fomentaram a avaliação de estabelecimentos de saúde contemplados com recursos financeiros quanto às dimensões de estrutura, processo e resultados, como o PMAQ-AB, em 2011. São exemplos o Programa de Requalificação da Infraestrutura das Unidades Básicas de Saúde do país (Requalifica UBS), de 2013, e o Programa Nacional de Avaliação de Serviços de Saúde (PNASS), de 2015. Desde a pesquisa de Donabedian^23^, entende-se que serviços de saúde que contam com estrutura adequada e têm equipes que seguem os protocolos e os princípios do seu modelo de atenção tendem a produzir melhores resultados na situação de saúde da população adscrita. Este entendimento pode embasar a tomada de decisão em prol da melhoria da saúde da população e reduzir desigualdades^24^. Em geral, as equipes têm mais autonomia para modificar seu processo de trabalho do que interferir na melhoria da infraestrutura, uma vez que isso implica em recursos financeiros que nem sempre estão disponíveis^24,25^.

Foi com essa intenção que as análises deste trabalho priorizaram estas características dos serviços e das equipes como essenciais para atenuar o efeito das desigualdades contextuais e dos seus reflexos em determinantes mais proximais da mortalidade, como a atenção pré-natal, o baixo peso ao nascer e as coberturas vacinais^12^.

À semelhança dos achados deste estudo, Hatisuka et al.^26^ também identificaram uma relação direta entre as menores TMI e as unidades com boa classificação de desempenho na avaliação do PMAQ, apesar da classificação utilizada não guardar relação direta com o cuidado dispensado à saúde materno-infantil, como as exposições selecionadas nestas análises.

Estudos realizados no Brasil, nos últimos anos, continuam a demonstrar o maior risco de mortalidade infantil entre crianças que apresentam baixo peso ao nascer^27^ e aquelas cujas mães tiveram um menor número de consultas de pré-natal^17^.

O retardo de crescimento intrauterino, os partos por cesariana e a prematuridade levam a uma maior ocorrência de baixo peso ao nascer, contribuindo para agravar a saúde do neonato e torná-lo mais vulnerável a doenças e infecções^28^.

Conforme preconizado pelo Ministério da Saúde, o pré-natal adequado permite o diagnóstico e o tratamento oportuno de fatores de risco e complicações durante a gestação, reduzindo ou modificando comportamentos de risco para amenizar a morbimortalidade da mãe e do feto^29^. É necessário garantir medidas de prevenção e proteção, como o início do acompanhamento até a 12ª semana de gestação, a realização de no mínimo seis consultas, duas rotinas de exames laboratoriais e testes rápidos para HIV, sífilis e hepatites B e C^30^.

Tanto o peso ao nascer quanto as consultas de pré-natal têm relação direta com a qualidade da assistência na gestação e no parto e podem refletir a condição socioeconômica e a morbidade materna, além de comportamentos maternos em relação aos cuidados de saúde^31^. Os achados deste estudo ratificam a importância da APS no acompanhamento e na adesão aos cuidados de pré-natal, do fortalecimento do vínculo com as usuárias e do planejamento familiar. Apesar dos avanços em ações, programas e políticas de saúde que incentivam os usuários na adesão aos cuidados em saúde e combate à pobreza, como o Bolsa Família^32^, é necessário investimento para qualificar o processo de trabalho das equipes e melhorar a infraestrutura dos serviços, a exemplo do PMAQ^26^.

Ainda no âmbito da APS, outra importante estratégia para a prevenção da mortalidade infantil refere-se às imunizações. O Programa Nacional de Imunizações (PNI), aprovado em 1973, busca proporcionar melhor qualidade de vida à população por meio da prevenção de doenças. As vacinas estimulam o sistema imunológico, protegendo a saúde e evitando incapacidades e mortes por agravos preveníveis, como a poliomielite, difteria, tétano, coqueluche, hepatite B, *haemophilus influenza* tipo b, sarampo, caxumba e rubéola, entre outras. A redução das coberturas vacinais tem potencial para a emersão de epidemias e aumento da mortalidade infantil^33,34^.

Os imunizantes podem ser considerados uma das principais tecnologias adjuvantes para a redução da mortalidade infantil, pois contribuíram para a erradicação de algumas doenças, agindo ainda na redução da gravidade dos sintomas e contribuindo para o aumento da expectativa de vida. Desde a descoberta do tratamento para a varíola, em 1796, por Edward Jenner, até o advento da vacina contra a covid-19, tem-se observado a eficácia, a efetividade, a segurança e a proteção dos imunizantes em nível individual e coletivo^35^.

Em relação à limitação deste estudo, destaca-se a utilização de bases de dados secundários, sem a possibilidade de analisar outros fatores associados à mortalidade infantil, como situação nutricional, adoecimento e utilização de serviços de saúde.

Neste estudo, evidenciou-se a relação entre a estrutura dos serviços de APS e o processo de trabalho das equipes com TMI, independente de fatores individuais, como as consultas de pré-natal, o peso ao nascer e a situação vacinal. Nesse sentido, o investimento na qualificação da atenção à saúde no âmbito da APS pode impactar na redução da TMI e na melhoria da atenção à saúde infantil.

Para a organização da APS e o planejamento das ações em saúde, é fundamental considerar as diferenças regionais, devido à heterogeneidade cultural, econômica e social, a fim de qualificar a atenção prestada, identificar áreas de risco e selecionar indicadores mais sensíveis a ações estratégicas.

Mesmo diante do declínio da TMI ao longo das últimas décadas, o efeito da pandemia de covid-19 pode implicar em piora dos indicadores de saúde, haja vista a possível redução na oferta de ações programáticas da APS para o atendimento da demanda no enfrentamento da pandemia.
